# Ultra-low dose protocol on photon-counting computed tomography as an alternative to radiographic shunt series in the diagnosis of mechanical ventriculoperitoneal shunt complications – an ex vivo phantom study for children and adults

**DOI:** 10.1007/s00234-026-03911-2

**Published:** 2026-02-03

**Authors:** Berk Yildirim, Aydin Demircioğlu, Raya Ocker-Serger, Laura Valentina Klüner, Marcel Drews, Sebastian Zensen, Hanna Styczen, Maximilian Schüßler, Yan Li, Benjamin Schröer, Denise Schönbeck, Christoph Mönninghoff, Thiemo Dinger, Philipp Dammann, Ulrich Sure, Helmut Schlattl, Patrizia Kunert, Michael Forsting, Cornelius Deuschl, Marcel Opitz, Denise Bos

**Affiliations:** 1 Institute of Diagnostic and Interventional Radiology and Neuroradiology, Essen University Hospital, Essen, Germany; 2 Clinic for Diagnostic and Interventional Radiology, University Hospital Heidelberg, Heidelberg, Germany; 3 Faculty of Medicine, University of Duisburg-Essen, Essen, Germany; 4 Department of Radiology, Neuroradiology and Nuclear Medicine, Johannes Wesling Medical Center, Minden, Germany; 5 Department of Neurosurgery, Essen University Hospital, Essen, Germany; 6 Department of Medical and Occupational Radiation Protection, Federal Office for Radiation Protection, Oberschleißheim, Germany; 7 Institute of Diagnostic and Interventional Radiology, University Hospital of Zurich, Zurich, Switzerland

**Keywords:** Shunt, ventriculoperitoneal, Computed tomography, Radiation protection

## Abstract

**Purpose:**

The standard modality for the diagnosis of ventriculoperitoneal (VP) shunt failure is the radiographic shunt series (RSS). However, ultra-low dose computed tomography (ULD-CT) may replace RSS. The aim of this study was to compare the radiation doses of RSS and ULD-CT in the diagnosis of mechanical shunt failure in human phantoms including pediatric phantoms and to further reduce the total CT dose by reducing the topogram radiation.

**Methods:**

Mechanical VP shunt complications were placed on human phantoms representing ages of 1, 5, 10 and 30 years. RSS and ULD-CT were performed on each phantom with different radiation doses of the topogram with varying tube currents (10, 20, 30, 40 and 50 mAs). Effective doses of RSS and ULD-CT were estimated by using conversion factors.

**Results:**

ULD-CT demonstrated lower effective doses than RSS in phantoms representing ages 5, 10 and 30 years, while successfully depicting all mechanical shunt complications. However, higher effective doses were assessed for ULD-CT scans of the 1-year phantom than RSS. The effective doses for RSS and ULD-CT (utilizing 10 mAs topograms), respectively, were estimated as follows: 1-year: 0.056 vs. 0.133 mSv; 5-year: 0.186 vs. 0.123 mSv; 10-year: 0.240 vs. 0.107 mSv; 30-year: 0.641 vs. 0.076 mSv.

**Conclusion:**

This study demonstrated ULD-CT as an alternative to RSS for the detection of mechanical VP shunt complications, reducing radiation doses in phantoms equivalent to 5 years of age and older while demonstrating the complications equally to RSS. This may be of clinical interest, especially in children due to the reduction of radiation risks.

**Supplementary information:**

The online version contains supplementary material available at 10.1007/s00234-026-03911-2.

## Introduction

 Hydrocephalus is a disorder where there is an excessive accumulation of cerebrospinal fluid (CSF) in the ventricles within the brain, potentially causing increased intracranial pressure and neurological symptoms. A commonly used treatment option of hydrocephalus is to connect the intracranial CSF space to the peritoneal cavity through a ventriculoperitoneal (VP) shunt, generating a flow of the excess CSF buildup from the brain into the abdominal cavity [1–3].

Despite being a widely practiced technique, there are various complications associated with VP shunt. These include infections and hemorrhage within the sites of the shunt course, ineffective or over-drainage of the CSF fluid, as well as mechanical complications such as shunt disconnection, dislocation and obstruction. Said complications lead to a significantly increased risk of hospital readmissions [4–6].

Radiological imaging is frequently utilized for the diagnosis of shunt failures. The main modalities that are considered in case of a mechanical failure are radiographic shunt series (RSS) and computed tomography (CT). RSS contains a set of radiographic images to illustrate the VP shunt course, generally consisting of skull, chest and abdomen projections in multiple planes. A CT scan of the head can be performed to assess a potential ventricular enlargement or hemorrhage in the brain in case of a suspected shunt failure [1, 7–10].

Radiation exposure due to radiological imaging is especially important in pediatric patients, as children are more susceptible to risks of ionizing radiation, mainly the development of malignancies [11–13]. Furthermore, previous literature reports significantly higher rates of mechanical VP shunt complications in children than in adults, resulting in an increased frequency of radiologic imaging examinations in the pediatric cohort [14, 15].

Several studies have been conducted on ultra-low dose CT (ULD-CT) as an alternative to RSS in the diagnosis of mechanical VP shunt complications, which demonstrated that ULD-CT can achieve lower radiation doses than RSS [7, 9, 10, 16–19].

Objective of the present study was to compare the diagnostic accuracy and radiation doses of RSS and ULD-CT on photon-counting computed tomography (PCCT) in the diagnosis of mechanical shunt failure in human phantom models including pediatric phantoms corresponding to various ages, as well as to further decrease radiation on PCCT within this setting by reducing the topogram tube current-time product.

## Materials and methods

### VP shunt preparation and study conduct

One VP shunt for RSS examinations, and two bilateral VP shunts (Sphera Duo shunt system and Integra VP shunt) for ULD-CT examinations were placed on the surface of each human phantom model. ATOM dosimetry phantoms (CIRS, Virginia, USA) corresponding to ages of 1, 5, 10 and 30 years (models 704, 705, 706, 701) were used for the study. The 1-year phantom weighed 10 kg at a height of 75 cm, the 5-year phantom 19 kg at 110 cm, the 10-year phantom 32 kg at 140 cm and the 30-year phantom 73 kg at 173 cm. The pediatric phantoms were not assigned to a gender, the adult phantom was classified as male.

**Fig. 1:**
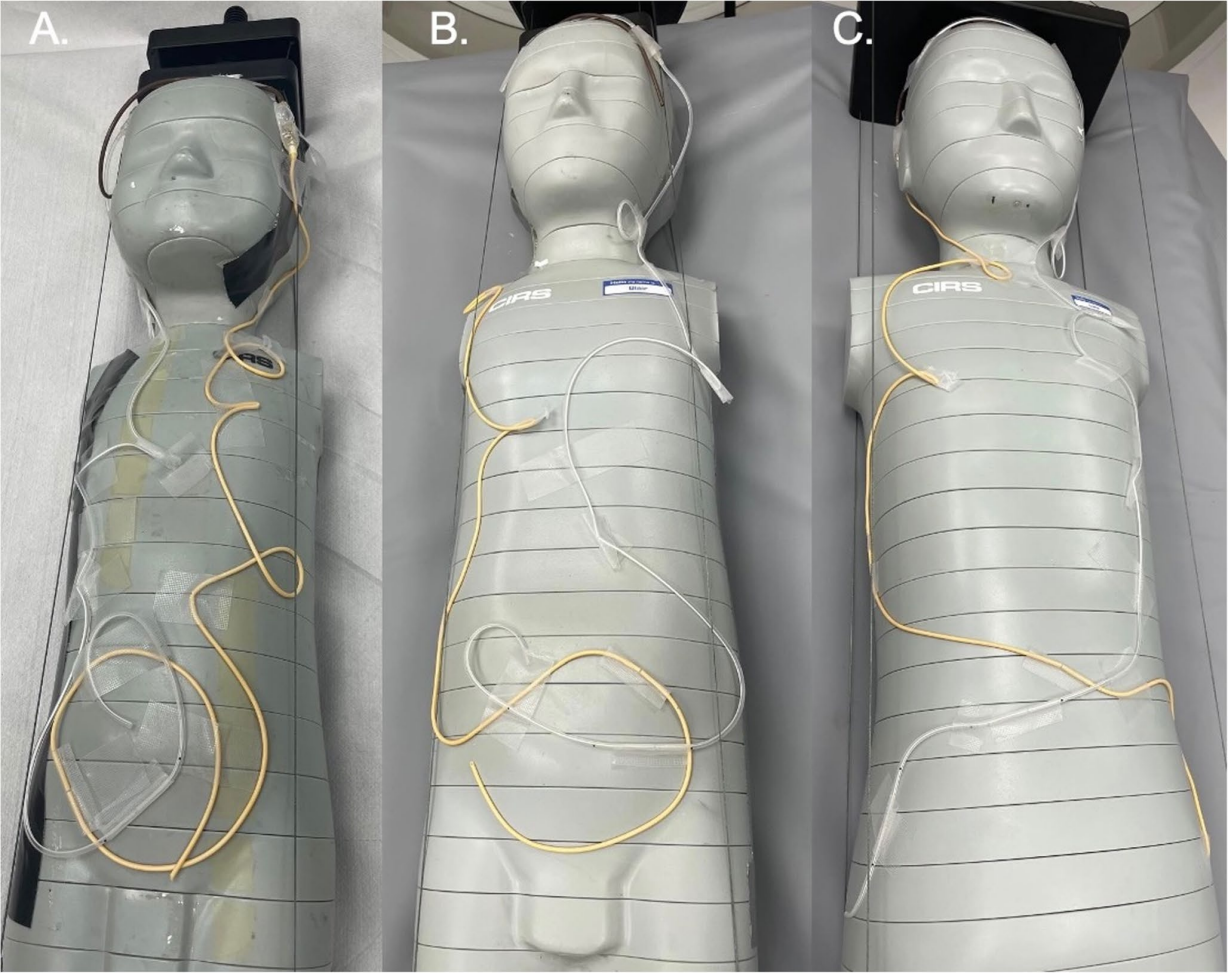
Pediatric human phantoms with implanted ventriculoperitoneal shunt complications. (**A**) 1-year phantom. (**B**) 5-year phantom. (**C**) 10-year phantom

VP shunt disconnections were introduced prior to the implantation on the phantoms. In addition to the sites of disconnection, mechanical shunt complications such as deformations in form of kinking and twisting, were created during the process of shunt placement on the phantoms (Fig. 1). RSS and ULD-CT scans for the demonstration of the complete shunt course were conducted on each phantom. A total of 29 mechanical shunt complications were implanted across all phantoms for the ULD-CT scans: eight disconnections, 21 deformations including 11 as kinking, 10 as twisting. A total of eight shunt complications were implanted across all phantoms for the RSS examinations: three disconnections, five deformations including four as kinking and one as twisting.

### RSS and ULD-CT protocols

Anteroposterior and lateral projections of the skull and abdomen, as well as anteroposterior projections of the chest were scanned in each RSS. This led to a total of five radiographic images on each phantom. The RSS acquisition parameters were set automatically according to the phantom body mass index (BMI) based on the standard institutional protocol. The radiographic examinations were conducted with Siemens Fluorospot Compact FD system (Siemens Healthineers, Erlangen, Germany).

CT scan ranges required to demonstrate the complete VP shunt course (head to pelvis) were determined according to topograms with differing radiation doses by varying the tube current-time products from 10, 20, 30, 40 to 50 mAs (at 100 kVp, one topogram at each mAs value). This was conducted to test by how much the total effective dose (ED) from a ULD-CT examination can be reduced through altering the topogram and whether this has any relevance on the consecutive CT scan. A separate CT scan was conducted following each topogram, which resulted in five topograms and five ULD-CT scans on each phantom. Thus, a total of 20 CT scans were conducted across all phantoms. CT studies were performed on the PCCT scanner Siemens NAEOTOM Alpha (Siemens Healthineers, Erlangen, Germany), utilizing the image quality level 1 (the lowest image quality level available on PCCT in our institution) with a volume weighted computed tomography dose index (CTDI_vol_) of 0.036 mGy, a tube voltage of 100 kV, a tube current-time product per slice of 2.5 mAs and a pitch of 0.6. The CTDI_vol_ for each topogram was approximately 0.01 mGy. Further acquisition parameters for RSS und ULD-CT on each phantom are shown in Table 1.

**Table 1:** Scan parameters and ED of RSS and ULD-CT. Total DLP for each ULD-CT scan was calculated in combination with the corresponding topogram prior to the scan. Total ED for ULD-CT was then calculated with the age-specific *k*-coefficients for DLP from Romanyukha et al. [24] based on the tissue weighting factors from ICRP 103 [25]. EDs for the various radiographic planes were calculated by utilizing the entrance dose conversion factors from Seidenbusch et al. [20–22], which were then added to attain the total ED from RSS for the phantom. CTDI_vol_ = volume weighted computed tomography dose index, DAP = dose-area product, DLP = dose-length product, ED = effective dose, RSS = radiographic shunt series, ULD-CT = ultra-low dose computed tomography, ap = anteroposterior, lat = lateral

Radiographic Shunt Series						Ultra-low Dose Computed Tomography					
Study	DAP [dGycm^2^]	Peak potential [kVp]	Tube current-time product [mAs]	ED [mSv]	Study based on different topograms with varying mAs		CTDI_vol_ [mGy]	Scan length [mm]	Topogram DLP [mGycm]	Total DLP [mGycm]	Total ED [mSv]
**Phantom 1 year**					**Phantom 1 year**						
Skull (ap)	0.321	69.8	3.7	0.005	10 mAs		0.036	477.5	0.16	2.07	0.133
Skull (lat)	0.136	69.8	1.7	0.003	20mAs		0.036	477.5	0.34	2.25	0.144
Chest	0.128	78.9	1.3	0.014	30 mAs		0.036	480.0	0.48	2.39	0.153
Abdomen (AP)	0.233	69.8	2.3	0.021	40 mAs		0.036	477.5	0.67	2.58	0.165
Abdomen (lat)	0.208	69.8	2.3	0.013	50 mAs		0.036	477.5	0.76	2.67	0.171
			Total ED:	**0.056**							
**Phantom 5 year**					**Phantom 5 year**						
Skull (ap)	0.492	69.8	5.7	0.003	10 mAs		0.036	615.0	0.19	2.65	0.123
Skull (lat)	0.301	69.8	3.4	0.002	20mAs		0.036	612.5	0.36	2.82	0.133
Chest	0.096	78.9	0.7	0.007	30 mAs		0.036	615.0	0.56	3.02	0.142
Abdomen (AP)	1.373	69.8	9.4	0.082	40 mAs		0.036	615.0	0.75	3.21	0.151
Abdomen (lat)	2.256	69.8	19.0	0.092	50 mAs		0.036	615.0	0.95	3.41	0.160
			Total ED:	**0.186**							
**Phantom 10 year**					**Phantom 10 year**						
Skull (ap)	0.798	69.8	5.1	0.003	10 mAs		0.036	752.5	0.22	3.24	0.107
Skull (lat)	0.501	69.8	3.4	0.003	20mAs		0.036	755.0	0.44	3.46	0.114
Chest	0.242	101.9	0.7	0.014	30 mAs		0.036	755.0	0.68	3.70	0.122
Abdomen (AP)	3.266	69.8	13.9	0.150	40 mAs		0.036	755.0	0.89	3.91	0.129
Abdomen (lat)	3.744	69.8	16.9	0.070	50 mAs		0.036	757.5	1.14	4.16	0.137
			Total ED:	**0.240**							
**Phantom 30 year**					**Phantom 30 year**						
Skull (ap)	2.025	69.8	15.1	0.008	10 mAs		0.036	842.5	0.24	3.64	0.076
Skull (lat)	4.394	71.3	11.9	0.011	20mAs		0.036	840.0	0.50	3.90	0.082
Chest	0.799	124.9	0.8	0.042	30 mAs		0.036	845.0	0.75	4.15	0.087
Abdomen (AP)	5.414	80.9	8.6	0.220	40 mAs		0.036	842.5	0.93	4.33	0.091
Abdomen (lat)	21.794	80.9	34.3	0.360	50 mAs		0.036	850.0	1.19	4.59	0.096
			Total ED:	**0.641**							

### Assessment of the radiation exposure

Following image acquisition parameters were documented for RSS: dose-area product (DAP), tube voltage peak, tube current-time product, patient size, focus-to-detector distance and field of view. Patient entrance doses were calculated using the formula:

$$\:E=\frac{DAP}{{\mathrm{A}}_{\mathrm{E}}}$$

where E is the entrance dose and A_E_ is the field size at the radiation entrance into the phantom. A_E_ was calculated using the following formula:

$$\:{A}_{E}={A}_{D}\times\:{\left(\frac{{r}_{D}-D}{{r}_{D}}\right)}^{2}$$

where A_D_ is the field of view, r_D_ is the focus-detector distance and D is the effective patient diameter.

EDs of the respective RSS projections were calculated by multiplying the corresponding entrance doses with the corresponding conversion factors from Seidenbusch et al. [20–22]. Total EDs were then calculated for each phantom by the addition of separate doses from the various RSS projections. Parameters used in the assessment of RSS doses are demonstrated in detail in Supplementary Table 1. It is worth mentioning that, according to the International Commission on Radiological Protection (ICRP) publication 121, the ED is not given as an absolute measure of radiation risk, but for a relative comparison of the doses from different imaging protocols [23].

Among the acquisition parameters of ULD-CT, scan lengths, dose-length products (DLP) and CTDI_vol_ were documented. Total ED was calculated by multiplying the total DLP with the age-specific *k*-factors from Romanyukha et al. for the chest on body computed tomography dose index (CTDI) phantoms [24] based on the tissue weighting factors from the ICRP publication 103 [25]:

$$\:{ED}_{total}=k\times\:{DLP}_{total}$$

Total DLP was calculated by adding the DLP of topogram to the DLP of the corresponding CT-scan:

$$\:{DLP}_{total}={DLP}_{topogram}+{DLP}_{CT}$$

### Assessment of mechanical shunt complications

Two blinded radiologists with at least 6 years of clinical experience assessed the mechanical shunt complications in RSS and ULD-CT scans of the 10 mAs topograms independently. A Likert scale ranging from 1 to 5 (1 = poor, 2 = limited, 3 = acceptable, 4 = good, 5 = very good) was used to evaluate the diagnostic confidence on each single mechanical shunt complication (disconnection, twisting, kinking) separately across all phantoms, as well as to evaluate how well the overall shunt course was depicted with RSS and ULD-CT separately on each phantom. In addition to the CT scans, volume rendering technique (VRT) and maximum projection intensity (MIP) reconstructions were performed for each VP shunt to depict an overview of the shunt course [26, 27] and assist the radiologists with identifying shunt complications.

### Statistical analysis

Mean values and standard deviations of the Likert scores by each reader were calculated for both imaging modalities. Cohen’s weighted kappa was used for measuring the inter-rater agreement on how well the complications were distinguishable and how well the overall shunt course was depicted in RSS and ULD-CT. R version 4.4.1 was utilized for the statistical analysis.

## Results

**Fig. 2:**
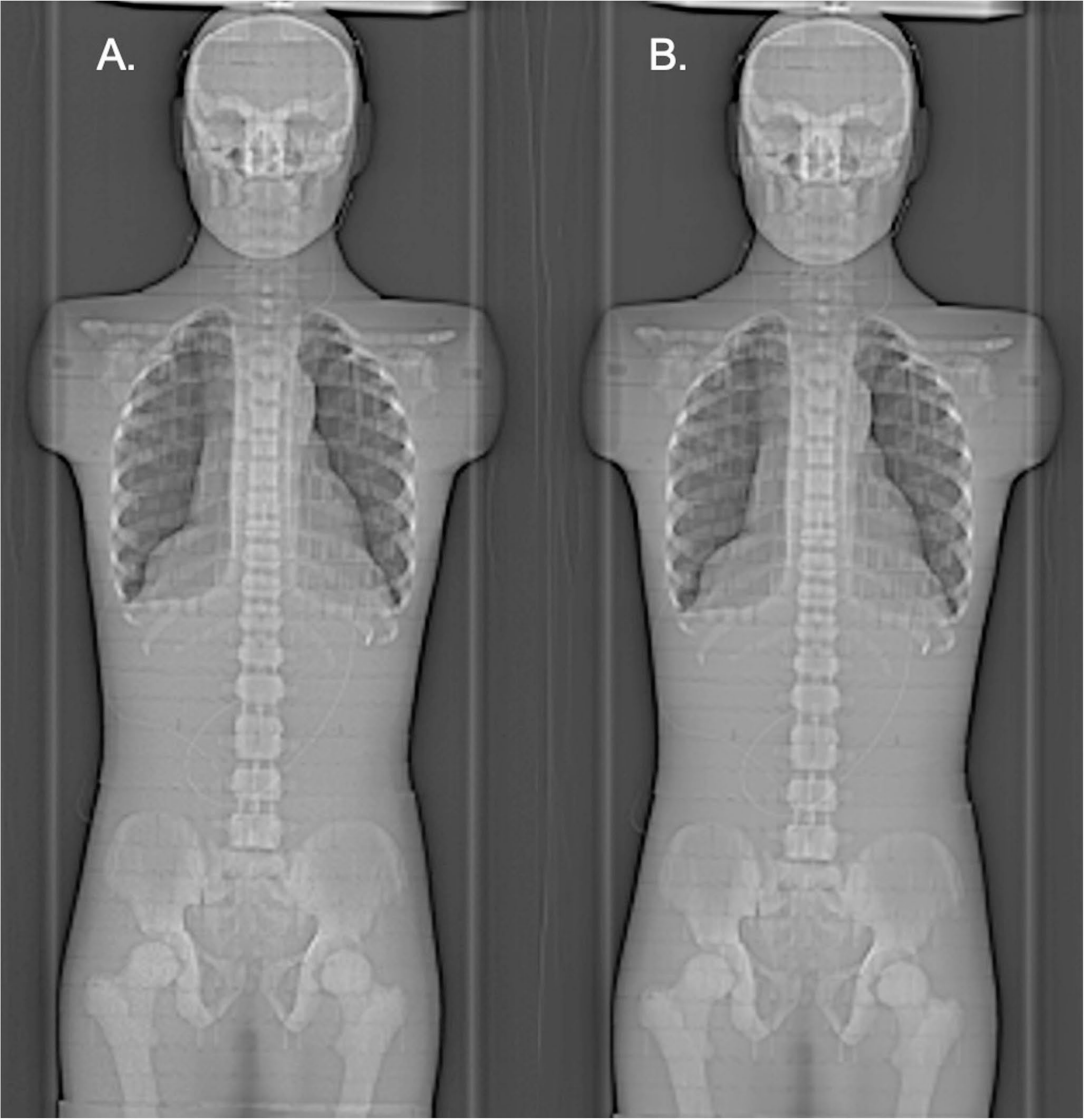
Adult phantom on topograms with differing tube currents. (**A**) 10 mAs topogram. (**B**) 50 mAs topogram. Both topograms depicted the complete ventriculoperitoneal shunt course for planning of the CT scan range

One RSS examination and five ULD-CT scans with prior topograms of differing tube current-time products (10 to 50 mAs) were conducted on each phantom. Lowering the tube current-time product of topograms prior to the CT scan did not have any influence on the demonstration of the shunt course in terms of prior planning of the CT scan length, as illustrated in Fig. 2.

### Radiation exposure

Total ED for ULD-CT increased with increasing topogram tube current-time product. ULD-CT achieved lower total ED for phantoms representing the ages of 5, 10 and 30 years in comparison to RSS. However, a higher total ED was delivered in the 1-year phantom with ULD-CT than with RSS. The EDs for RSS and ULD-CT (using the lowest dose by utilizing 10 mAs topograms), respectively, were as follows: 1-year: 0.056 vs. 0.133 mSv; 5-year: 0.186 vs. 0.123 mSv; 10-year: 0.24 vs. 0.107 mSv; 30-year: 0.641 vs. 0.076 mSv. Total EDs of the ULD-CT examinations with higher topogram tube current-time products ranging from 20 to 50 mAs, as well as further scan parameters indicative of the radiation exposure such as DAP for RSS and total DLP for ULD-CT, are depicted in Table 1.

**Fig. 3:**
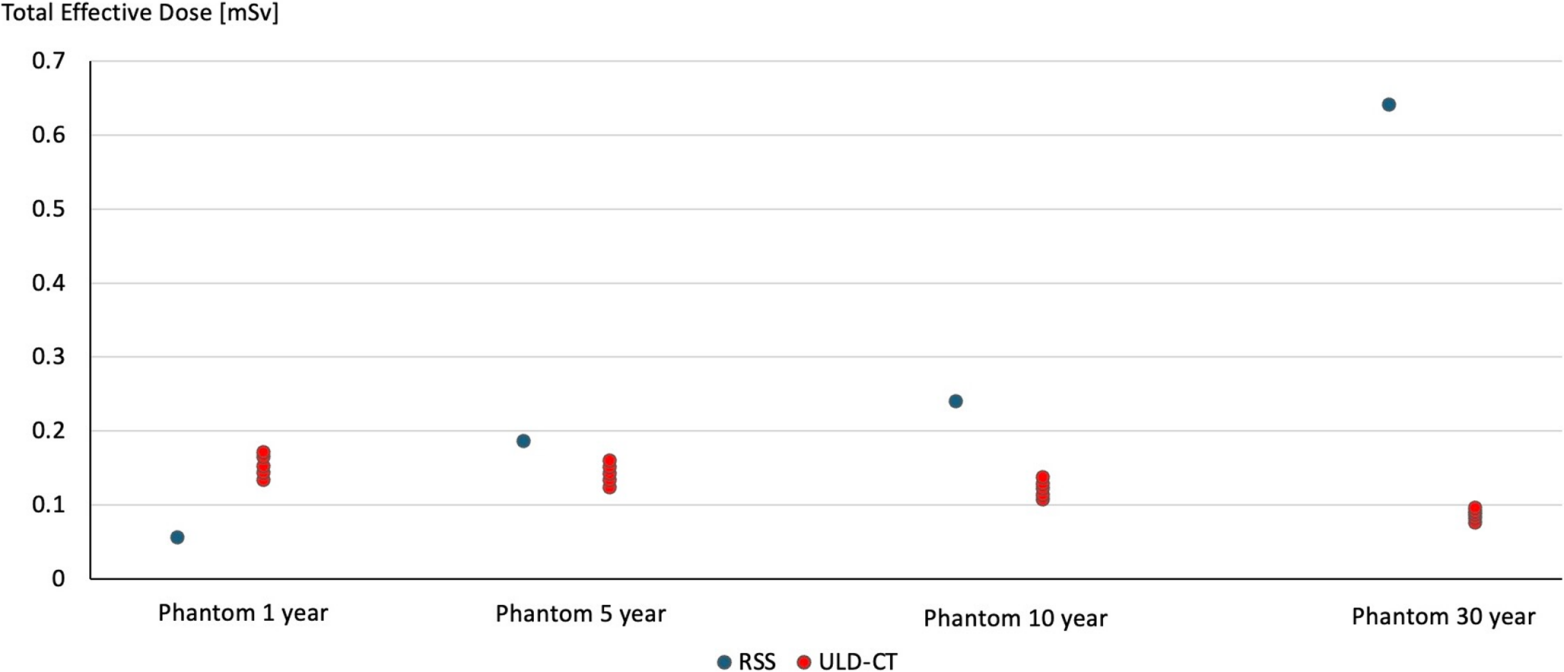
Comparison of total EDs for RSS and ULD-CT. Total EDs for ULD-CT were calculated by adding the doses from each scan with the corresponding topogram dose (10, 20, 30, 40, and 50 mAs). Total ED for each RSS was calculated by adding the doses from various radiographic planes of the same series. ED = effective dose, RSS = radiographic shunt series, ULD-CT = ultra-low dose computed tomography

Comparison of the total ED caused by RSS and ULD-CT for each phantom is further demonstrated in Fig. 3.

With increasing corresponding age of the phantom, total ED increased for RSS and decreased for ULD-CT. Furthermore, the benefit in radiation protection through the application of ULD-CT in comparison to RSS increased with increasing corresponding phantom age: Ratio of the total ED of ULD-CT (with 10 mAs topogram) to the total ED of RSS for the 1-year phantom was 238%, for the 5-year phantom was 66%, for the 10-year phantom was 45% and for the 30 year-phantom was 12%.

**Fig. 4:**
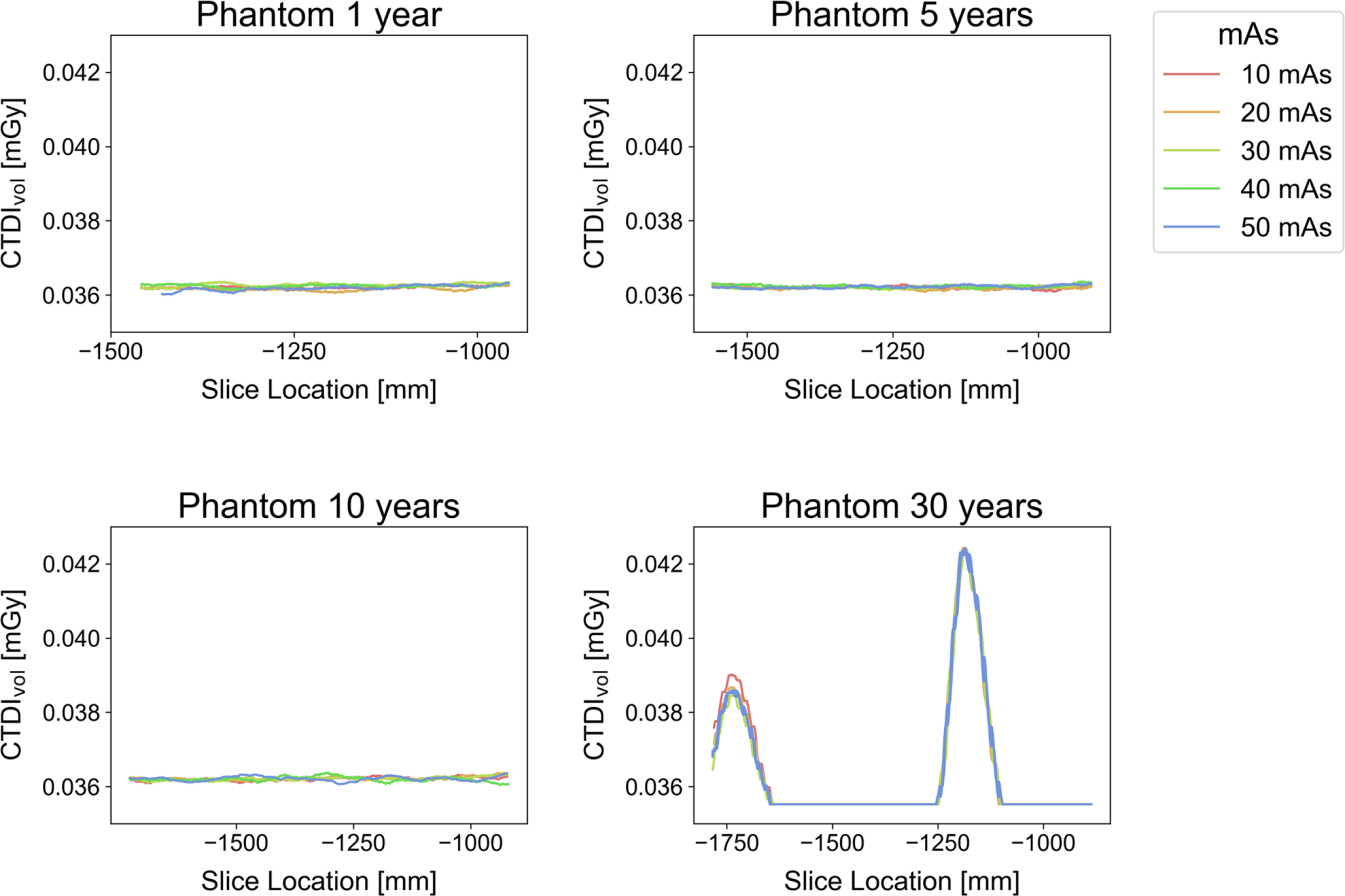
Dose modulation within the CT examinations conducted with topogram tube current-time products between 10 and 50 mAs in each phantom. X-axis represents the slice location within the phantom. As the value increases from left to right, the slice location moves down from the phantom head to pelvis. Y-axis represents the CTDI_vol_ value at the corresponding slice location. CTDI_vol_ = volume weighted computed tomography dose index

The percentages of the ED resulting from 10 to 50 mAs topograms within the total ED of the ULD-CT examination, respectively, were as follows: 1-year: 8% and 29%, 5-year: 7% and 28%, 10-year: 7% and 27%, 30-year: 7% and 26%. Reducing the topogram current-time product from 50 to 10 mAs resulted in the following reduction of the total ED from ULD-CT examinations: 1-year: 0.171 to 0.133 mSv (reduction by 22%); 5-year: 0.160 to 0.123 mSv (reduction by 23%); 10-year: 0.137 to 0.107 mSv (reduction by 22%); 30-year: 0.096 to 0.076 mSv (reduction by 21%). The dose modulation in CTDI_vol_ within the CT scan was similar regardless of the topogram, while no relevant modulation was observed in the pediatric phantoms, only in the adult phantoms in the shoulder and hip region, Fig. 4.

### Detection of the mechanical shunt complications

All mechanical shunt complications were detected accurately by both readers in RSS and ULD-CT examinations. Reader 1 rated the diagnostic confidence of how well the complications were distinguishable as 4.75 (± 0.71) and how well the overall shunt course was depicted as 4.75 (± 0.5) in RSS, whereas 4.64 (± 0.81) and 4.5 (± 0.58) in ULD-CT, respectively. Reader 2 rated the diagnostic confidence of how well the complications were distinguishable as 4.75 (± 0.71) and how well the overall shunt course was depicted as 4.75 (± 0.5) in RSS, whereas 4.73 (± 0.47) and 4.5 (± 0.58) in ULD-CT, respectively. The inter-observer agreement measured by Cohen’s weighted kappa equaled to 1 for both how well the complications were distinguishable and how well the overall shunt course was depicted in RSS. In ULD-CT, the inter-observer agreement for how well the overall shunt course was depicted also equaled to 1, however, it measured 0.5 (95% CI [0.27, 0.71]) for how well the complications were distinguishable.

**Fig. 5:**
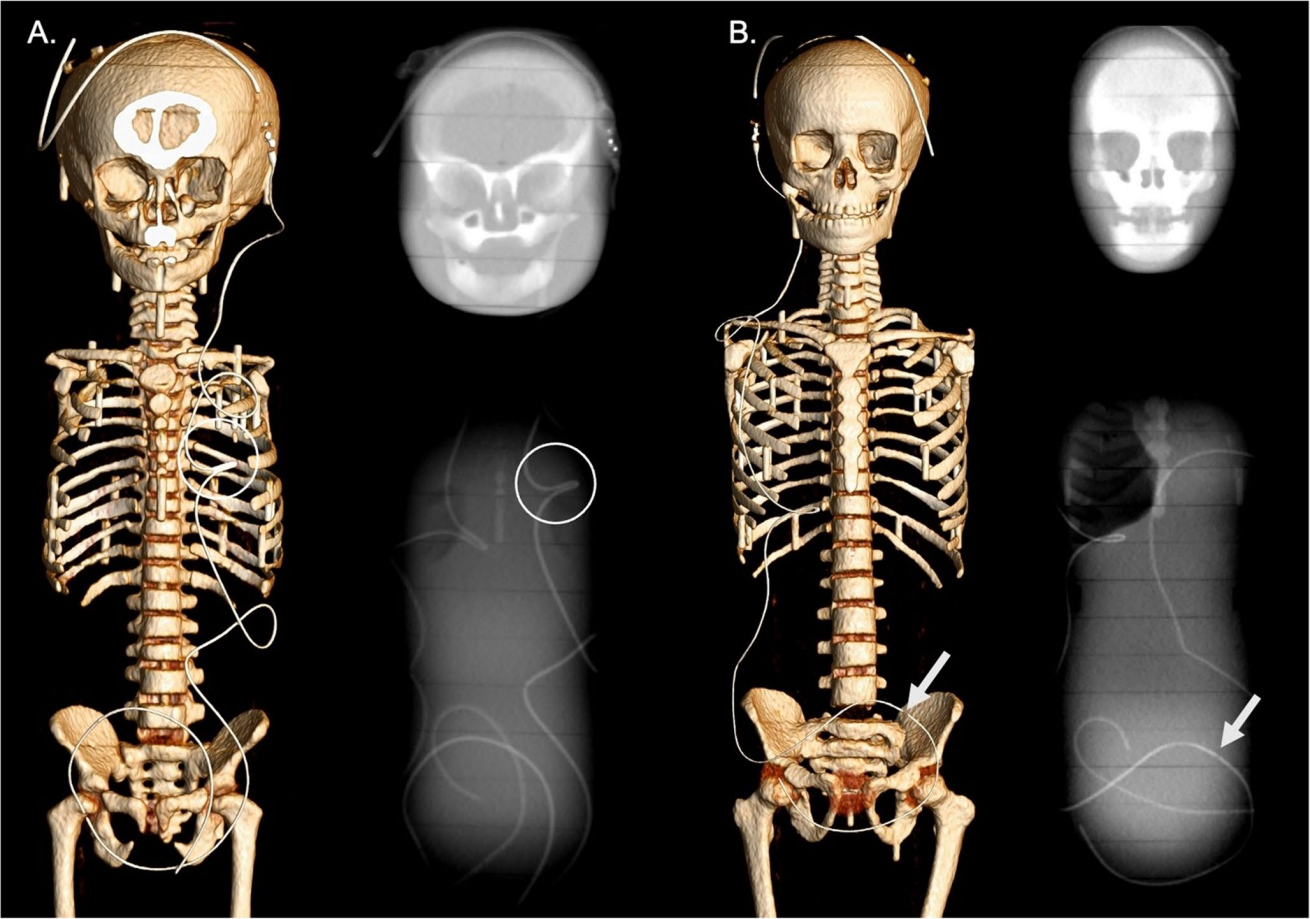
Maximum projection intensity and volume rendering technique reconstructions of ultra-low dose computed tomography scans on different phantoms. (**A**) On 1-year phantom. Kinking of the shunt catheter is marked with circles. (**B**) On 5-year phantom. Disconnection of the shunt catheter is marked with arrows

Examples of mechanical shunt complications in ULD-CT with MIP and VRT reconstructions are presented in Fig. 5.

## Discussion

To our knowledge, this is the first publication that compared RSS und ULD-CT using PCCT in the diagnosis of mechanical VP shunt complications in both pediatric and adult human phantoms, as well as the first study to test different topogram tube current-time product levels within the said setting to lower the total ED of ULD-CT examinations. In the present study, ULD-CT achieved lower estimated total ED than RSS for the human phantoms representing ages 5 years and above, while maintaining excellent sensitivity and high diagnostic confidence for mechanical VP shunt complications, which is in correlation with previous ex vivo studies [7, 17–19].

Despite failing to demonstrate lower ED on the 1-year phantom than RSS, ULD-CT lead to an increasing benefit in reduction of radiation exposure with increasing corresponding phantom age compared to RSS. Even on the 5-year phantom ULD-CT produced approximately 65% of the ED of RSS. The total ED from ULD-CT remained around 0.1 mSv across all the phantoms, whereas the ED from RSS increased noticeably with increasing corresponding phantom age. This is due to image quality level 1 being the lowest level achievable on PCCT at our institution with a consistent CTDI_vol_ across all phantoms, while RSS acquisition parameters were adjusted according to the physical phantom characteristics.

Reducing the topogram dose by lowering the tube current-time product led to a further radiation dose reduction by over 20% and did not seem to have any visible impact on the CT scan or the dose modulation. Furthermore, no relevant dose modulation in CTDI_vol_ was observed in the pediatric phantoms possibly due to the very low dose level, small phantom sizes and rather circular body cross-sections. Only in the adult phantom, dose modulation was seen in the shoulder and hip area. Thus, an important aspect of the discussion is whether to keep the topogram dose as low as possible, or to skip it completely in children if a sufficient scan range can be ensured for capturing the complete VP shunt course. This could lead to a further dose reduction of approx. 20–30%. However, previous literature suggests that CT topograms can deliver additional clinically relevant information on the patient, such as bone fractures and metastases [28, 29]. A 2024 published study even determined that CT topograms combined with cranial CT can be sufficient to assess the complete VP shunt course [30]. It is worth mentioning that motion artifacts hinder the evaluation of CT series. This aspect is especially important in younger pediatric patients, who may require anesthesia for ULD-CT examinations.

Apart from having a low number of scan series, there were several other limitations to this work. This was a preclinical phantom study including pediatric and adult human phantoms corresponding to four different ages. Due to the low BMI of the phantoms, they were not representative of patient cohorts with higher BMI, who may receive higher ED during radiological imaging. Furthermore, the impact of different genders was not considered during the assessment of EDs, as the pediatric phantoms were not assigned to a specific gender and the adult phantom was defined as male.

A further limitation was variation of the shunt complications in type and number between the two imaging modalities, with one shunt system for RSS and two different shunts for ULD-CT. As the RSS represents the current standard of VP shunt imaging when a mechanical shunt complication is suspected, our focus was to thoroughly assess the diagnostic confidence of ULD-CT in addition to the radiation exposure, hence the higher number of complications in ULD-CT. An ideal comparison of the two modalities within the context of mechanical shunt complications is difficult, as these complications were assessed by blinded radiologists on both imaging modalities. Even with the same number of complications, these have to be created at different shunt sites so that the radiologists cannot memorize the shunt complications by type and location prior to the assessment of the other imaging modality.

Because the VP shunts were placed onto and not into the phantoms, the ULD-CT depiction of the shunt course on the chest and the abdomen was partially impaired, whereas the detection of shunt complications along the neck and periclavicular region in ULD-CT may have become easier. These factors introduced potential bias to the comparison of the two imaging modalities in terms of diagnostic confidence and ED.

Furthermore, pathologies associated with a ventriculoperitoneal shunt, such as pseudocysts, hematomas or infections, could not be recreated in the present phantom design for diagnostic assessment. Another important aspect of the shunt course assessment in ULD-CT is whether the VP shunt ends inside the peritoneal cavity, which could not be analyzed due to the placement of the shunts on the phantoms. Finally, there is currently just one photon-counting CT of one vendor available, and thus the examined impact of the topogram dose on the reconstructed image quality could be vendor-specific. It might be different for forthcoming photon-counting CTs of other vendors.

As mentioned in previous literature, a direct comparison on the radiation exposure of X-rays and CT imaging is challenging [7, 9, 31]. In this study, EDs of the two imaging modalities were acquired through different methods: EDs of RSS by utilizing the entrance dose conversion factors from the publications of Seidenbusch et al. [20–22] and EDs of ULD-CT through the age-specific *k*-coefficients for DLP according to Romanyukha et al. [24] based on the tissue weighting factors from ICRP 103 [25]. Further scan parameters indicative of the radiation exposure are applicable to only one of the modalities, such as DAP for X-rays and DLP, CTDI_vol_ for ULD-CT. As the *k*-coefficients for the chest on CTDI body phantoms from Romanyukha et al. were used for our calculations, the total ED from ULD-CT in our study was overestimated, because the *k*-coefficients for CT scans of the head and the abdomen are lower with the exception on adult phantom (0.021 for the chest vs. 0.022 for the abdomen) [24] and our ULD-CT scans included a region starting from the head and ending at the pelvis. However, in the context of pediatric patients, it is better to overestimate the ED of an alternative imaging method than to underestimate. Albeit it can be expected that the EDs for ULD-CT are smaller than presented in this work, and thus also for the 1-year phantom the difference between ULD-CT and RSS would be smaller.

For the 1-year and 5-year phantom, instead of utilizing a single anteroposterior projection of the chest and abdomen for the demonstration of the majority of the VP shunt course within a single radiographic image, same projections were used as for the 10-year and adult phantom including separate projections of the chest and abdomen. This may have led to higher ED from RSS than with a single anteroposterior projection.

The present study did not analyze the impact of arm position during ULD-CT examinations on the ED, as the phantoms used in this study did not possess upper extremities. Because *k*-coefficients were utilized for the transparency of ULD-CT dose estimations, the study assumes that the patient is capable of changing their arm positioning to reduce radiation exposure and increase image quality. In a clinical setting, adjustment of the arm positioning for CT examinations may not be possible due to various reasons, such as neurological impairment or fractures of the upper extremities that are being treated at the time of the examination.

Although multiple ex vivo studies have demonstrated the potential role of low-dose CT examinations in the diagnosis of mechanical VP shunt complications [7, 17–19], there is still need for further evidence on comparison of RSS and ULD-CT in the clinical setting. Two retrospective clinical studies demonstrated lower estimated ED achieved by low-dose CT compared to RSS and a statistically significant difference in the estimated EDs between the two modalities [32, 33]. In the 2023 published pediatric clinical study of Kesheh et al., although ULD-CT enabled the detection of additional findings concerning VP shunt course and lowered the total time required for radiologic imaging in a hospital setting, it caused higher estimated mean ED than RSS [34]. Currently, ULD-CT requiring PCCT technology is an important limitation to the generalizability of ULD-CT in contrast to RSS.

Further research is required in a clinical setting on the comparison of ULD-CT and RSS in diagnosis of mechanical VP shunt malfunction, especially to analyze the diagnostic accuracy. Clinical trials should also include or be focused on pediatric patients, as dose reduction is of greater importance in children. It would also be of great interest to investigate how low we can go with the radiation dose through further technological advances.

## Conclusion

ULD-CT may have the potential to replace RSS as the standard imaging modality for the diagnosis of mechanical VP shunt dysfunction, potentially offering lower radiation exposure than RSS with similar diagnostic confidence. Further in vivo clinical studies are needed to further analyze the diagnostic accuracy.

## Supplementary information

Below is the link to the electronic supplementary material.

ESM 1 (DOCX 23.4 KB)

## Data Availability

No datasets were generated or analysed during the current study.
