# Supplementary material for: Ultra-low dose protocol on photon-counting computed tomography as an alternative to radiographic shunt series in the diagnosis of mechanical ventriculoperitoneal shunt complications – an ex vivo phantom study for children and adults
Source: Neuroradiology. 2026 Feb 3;68(7):1799–810. doi: 10.1007/s00234-026-03911-2 (PMC13407748; doi:10.1007/s00234-026-03911-2)
Supplement: Supplementary file 1 — (DOCX 23.4 KB) [file 234_2026_3911_MOESM1_ESM.docx]

Ultra-low dose computed tomography as an alternative to radiographic shunt series in the diagnosis of mechanical ventriculoperitoneal shunt complications – an ex vivo phantom study for children and adults

**Authors:** Berk Yildirim^1^, Aydin Demircioğlu^1^, Raya Serger^1^, Laura Valentina Klüner^1^, Marcel Drews^1^, Sebastian Zensen^1^, Hanna Styczen^1^, Maximilian Schüßler^1^, Yan Li^1^, Benjamin Schröer^2^, Denise Schönbeck^3^, Christoph Mönninghoff^1,3^, Thiemo Dinger^4^, Philipp Dammann^4^, Ulrich Sure^4^, Helmut Schlattl^5^, Patrizia Kunert^5^, Michael Forsting^1^, Cornelius Deuschl^1^, Marcel Opitz^1,†^, Denise Bos^1,6,†^.

**Author affiliations:**

^1^Institute of Diagnostic and Interventional Radiology and Neuroradiology, University Hospital Essen, University of Duisburg-Essen, Essen, Germany.

^2^Faculty of Medicine, University Duisburg-Essen.

^3^Deparment of Neuroradiology, Johannes Wesling Medical Center, Minden, Germany.

^4^Department of Neurosurgery, University Hospital Essen, University of Duisburg-Essen, Essen, Germany.

^5^Department of Medical and Occupational Radiation Protection, Federal Office for Radiation Protection (BfS), Oberschleißheim, Germany.

^6^Institute of Diagnostic and Interventional Radiology, University Hospital Zurich, Zurich, Switzerland.

^†^These authors contributed equally to this work.

**Corresponding author:**

Berk Yildirim

Institute of Diagnostic and Interventional Radiology and Neuroradiology,

University Hospital Essen,

Hufelandstrasse 55,

45147 Essen, Germany.

Tel.: (+)49-151-42459537

E-mail: [Berk.Yildirim@uk-essen.de](mailto:Berk.Yildirim@uk-essen.de)

ORCID ID: 0009-0009-8533-6016

**Journal name:** Neuroradiology.

**Supplementary Table 1:** Parameters for entrance dose calculation and entrance doses of radiographic shunt series. ap = anteroposterior, lat = lateral.

| Study | Field of view [cm^2^] | Effective patient diameter [cm] | Focus-detector distance [cm] | Entrance dose [mSv] |
| --- | --- | --- | --- | --- |
| **Phantom 1 year** |  |  |  |  |
| Skull (ap) | 19.60 x 25.82 | 14.6 | 114.9 | 0.083 |
| Skull (lat) | 17.21 x 28.15 | 12.7 | 114.9 | 0.036 |
| Chest | 20.55 x 22.31 | 11.5 | 114.9 | 0.035 |
| Abdomen (ap) | 21.86 x 28.04 | 11.8 | 114.9 | 0.047 |
| Abdomen (lat) | 18.29 x 30.57 | 13.7 | 114.9 | 0.048 |
|  |  |  |  |  |
| **Phantom 5 year** |  |  |  |  |
| Skull (ap) | 16.92 x 29.62 | 15.9 | 114.9 | 0.132 |
| Skull (lat) | 18.71 x 28.35 | 13.5 | 114.9 | 0.073 |
| Chest | 25.86 x 26.42 | 13.7 | 114.9 | 0.018 |
| Abdomen (ap) | 27.49 x 30.54 | 13.0 | 114.9 | 0.208 |
| Abdomen (lat) | 20.19 x 34.03 | 17.5 | 114.9 | 0.457 |
|  |  |  |  |  |
| **Phantom 10 year** |  |  |  |  |
| Skull (ap) | 21.99 x 37.11 | 16.9 | 107.3 | 0.138 |
| Skull (lat) | 21.51 x 35.61 | 14.0 | 107.3 | 0.087 |
| Chest | 30.57 x 34.24 | 16.2 | 107.3 | 0.032 |
| Abdomen (ap) | 29.62 x 40.94 | 14.7 | 107.3 | 0.362 |
| Abdomen (lat) | 29.02 x 39.01 | 18.4 | 107.3 | 0.444 |
|  |  |  |  |  |
| **Phantom 30 year** |  |  |  |  |
| Skull (ap) | 20.48 x 38.20 | 20.8 | 115.0 | 0.386 |
| Skull (lat) | 30.60 x 33.35 | 15.0 | 115.0 | 0.569 |
| Chest | 43.17 x 34.03 | 22.4 | 115.0 | 0.084 |
| Abdomen (ap) | 34.03 x 43.20 | 21.2 | 115.0 | 0.554 |
| Abdomen (lat) | 34.03 x 43.20 | 27.6 | 115.0 | 2.567 |
